# Long-term feeding ecology and habitat use in harbour porpoises *Phocoena phocoena *from Scandinavian waters inferred from trace elements and stable isotopes

**DOI:** 10.1186/1472-6785-7-1

**Published:** 2007-01-17

**Authors:** Michaël C Fontaine, Krystal A Tolley, Ursula Siebert, Sylvie Gobert, Gilles Lepoint, Jean-Marie Bouquegneau, Krishna Das

**Affiliations:** 1 MARE Centre – Laboratory for Oceanology, University of Liège, B6c, Liège Sart Tilman B-4000, Belgium; 2 CBGP-INRA (Centre de Biologie et de Gestion des Populations). Campus international de Baillarguet CS 30016, 34988 Montferrier-sur-Lez cedex, France; 3 Molecular Ecology and Evolution Program, Kirstenbosch Research Centre, South African National Biodiversity Institute, P/Bag X7, Claremont 7735, South Africa; 4 Institute of Marine Research, PO Box 1870, Nordnes, 5024, Bergen, Norway; 5 Forschungs- und Technologie Zentrum Westküste, Universität Kiel, Werfstraße 6, 25761 Büsum, Germany

## Abstract

**Background:**

We investigated the feeding ecology and habitat use of 32 harbour porpoises by-caught in 4 localities along the Scandinavian coast from the North Sea to the Barents Sea using time-integrative markers: stable isotopes (δ^13^C, δ^15^N) and trace elements (Zn, Cu, Fe, Se, total Hg and Cd), in relation to habitat characteristics (bathymetry) and geographic position (latitude).

**Results:**

Among the trace elements analysed, only Cd, with an oceanic specific food origin, was found to be useful as an ecological tracer. All other trace elements studied were not useful, most likely because of physiological regulation and/or few specific sources in the food web. The δ^13^C, δ^15^N signatures and Cd levels were highly correlated with each other, as well as with local bathymetry and geographic position (latitude). Variation in the isotopic ratios indicated a shift in harbour porpoise's feeding habits from pelagic prey species in deep northern waters to more coastal and/or demersal prey in the relatively shallow North Sea and Skagerrak waters. This result is consistent with stomach content analyses found in the literature. This shift was associated with a northward Cd-enrichment which provides further support to the Cd 'anomaly' previously reported in polar waters and suggests that porpoises in deep northern waters include Cd-contaminated prey in their diet, such as oceanic cephalopods.

**Conclusion:**

As stable isotopes and Cd provide information in the medium and the long term respectively, the spatial variation found, shows that harbour porpoises experience different ecological regimes during the year along the Scandinavian coasts, adapting their feeding habits to local oceanographic conditions, without performing extensive migration.

## Background

Harbour porpoises (*Phocoena phocoena*) are among the smallest cetaceans, and are widely distributed in cold waters of the temperate and sub-arctic northern hemisphere [1]. They occur primarily over continental shelves, although some individuals are occasionally found in deeper waters [2]. Throughout their distribution, harbour porpoises are vulnerable to mortality in commercial gillnet fisheries [3]. The development and application of new technologies, including time-depth recorders and satellite geolocation, have substantially increased our knowledge of habitat use by marine mammals [4]. However, until now, the application of these techniques to harbour porpoises remains limited and geographically localized [5-7]. While these studies have revealed remarkable aspects of porpoise movements and diving capabilities, they can hardly be extrapolated from one area to another and they do not directly address feeding habits.

As a small endothermic predator with limited energy storage capacity, it is assumed that harbour porpoises must feed frequently without prolonged periods of fasting [8]. Consequently, their distribution and movements should essentially follow those of their prey, as suggested by porpoise satellite tracking [7]. The feeding ecology of the harbour porpoise has been the subject of numerous studies in different parts of its range, almost exclusively from examination of prey remains in stomach contents (reviewed in [9]). Theses studies showed primarily piscivorous feeding habits, and indicated considerable spatial and temporal variation in the diet of the species. However, while analyses of prey remains from stomach contents provide essential insights into the diet of marine mammals, they may not reveal long-term feeding habits, the use of patchily distributed, locally abundant food resources, and the relative importance of particular prey species to an animal's diet [10].

Complementary approaches based on the analysis of elemental composition of tissue are increasingly used as time-integrative markers to delineate feeding ecology of species, habitat use, and population identity [10-13]. The stable isotope composition of animal tissues reflects the isotopic composition of their diet, following the maxim "You are what you eat" [14]. Isotopic studies have revealed new details regarding the foraging ecology of marine vertebrates, such as trophic position, reliance on nearshore or offshore food sources, the relative importance of different prey species, and interspecific or intraspecific resource partitioning [11-13]. Carbon isotope ratios (^13^C/^12^C) display little or no change in the abundance between trophic levels (~1‰) following the primary producer to primary consumer link [15]. As such, isotopes of this chemical element are useful to discriminate the origin of primary productivity in simple systems where isotopically distinct sources are present (*e.g*., phytoplankton *vs*. kelp forest) [15-18]. In the marine environment, benthic and coastal food webs are enriched in ^13^C relative to pelagic food webs. Stable carbon isotopes can thus provide valuable information about preferred habitats and carbon sources. On the other hand, the stable nitrogen isotope ratios (^15^N/^14^N) show a systematic enrichment with trophic level, a relatively constant effect of typically 3–4‰ per level [19] which can be used to model the position of consumers in the marine food webs. These ecological tracers, together with more conventional dietary approaches, can thus provide complementary information on trophic relationships.

The trace elements have also been tentatively used as ecological tracers [20-24]. They can be classified in two main categories: *essential elements *(*e.g*., selenium (Se), zinc (Zn), copper (Cu), iron (Fe)) which play key roles in many biochemical pathways, generally as co-factors for enzymes, and are under strong homeostatic regulation; *non-essential elements *(*e.g*., mercury (Hg), cadmium (Cd)) which have no known biochemical functions and may interfere with essential elements [25]. Marine mammals, as top predators, accumulate trace elements in their tissues from their environment, chiefly *via *their food [26]. One could therefore expect that animals foraging in habitats or on geochemically distinct prey species may reflect those differences in characteristic elemental composition of their tissues. However, one could also assume that essential elements under strong physiological regulation would be of limited value as dietary biomarkers compared to the non-essential elements.

On the basis of these assumptions about isotopic and elemental behaviours in ecosystems, increasing attempts have been made to use differences in the compositions of environmentally acquired elements to evaluate population identity and discreteness in marine vertebrates [12,21,22,27,28]. This approach is based on the rationale that groups of animals exploiting geochemically different habitats should have tissues reflecting those differences in characteristic element or isotope compositions ("fingerprints"). If such differences are found in element/tissue combinations which are not subject to rapid turnover, they may indicate the existence of "ecological segregation".

**Figure 1:**
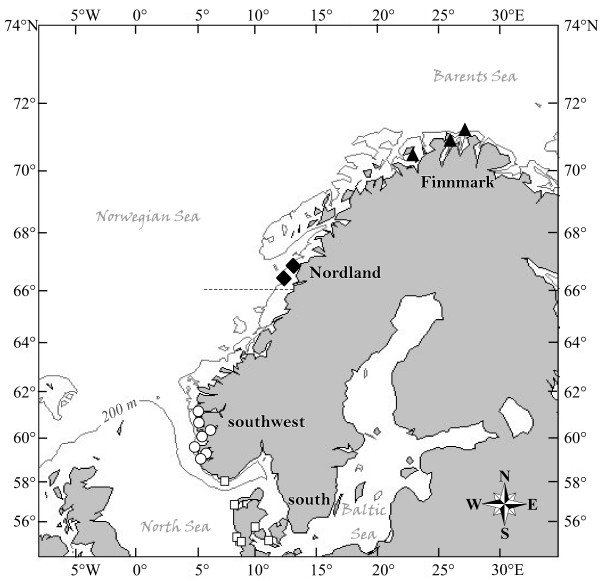
**Sampling locations of harbour porpoises along the Scandinavian coast**. Samples come from four regions namely southern waters (West Agder and Danish waters, white squares); the southwest coast of Norway (North Sea; white circles), Nordland (Norwegian Sea; black diamonds), and Finnmark (Barents Sea; black triangles). The dotted line (at 66°N) indicates the separation of the two putative population (North Sea and the North Norway/Barents Sea) as defined by the International Whaling Commission [62].

In Norwegian and adjacent coastal waters, stomach content analyses [29] indicated that harbour porpoise diet varied between neighbouring areas and that this variation in diet composition could be related to local habitat characteristics, and especially the bathymetry. We can expect that, if this ecological variation is persistent over time, it should have a strong impact on time-integrative ecological markers, such as stable isotope and trace elements. In this study, we examined the spatial variation in composition of stable isotopes (δ^13^C, δ^15^N) and of six trace elements (Zn, Cu, Fe, Se, Hg, Cd) in harbour porpoises of relatively similar age (Table 1), collected along the Scandinavian coast from the North Sea to the Barents Sea, with regards to the local bathymetry and the latitude (Figure 1). We have also assessed the practicality of using essential and non-essential trace elements as tracers to characterize variation in the porpoise feeding ecology and habitat use by this species.

**Table 1:** Means and ranges of harbour porpoise ages (in years) and local bathymetry (m.) within the 100 km radius around the collection sites.

	Region	n	Mean ± SD	Range
Age	southern	12	1.6 ± 0.3	0.5 – 3.0
	southwest	9	3.1 ± 0.3	1.5 – 5.0
	Nordland	7	4.5 ± 1.4	1.5 – 12.0
	Finnmark	3	2.3 ± 0.3	2.0 – 2.9
Bathymetry	southern	13	53 ± 65	12 – 221
	southwest	9	157 ± 23	104 – 189
	Nordland	7	210 ± 7	200 – 215
	Finnmark	3	226 ± 37	187 – 262

n = number of samples, SD = standard deviation

## Results

**Figure 2:**
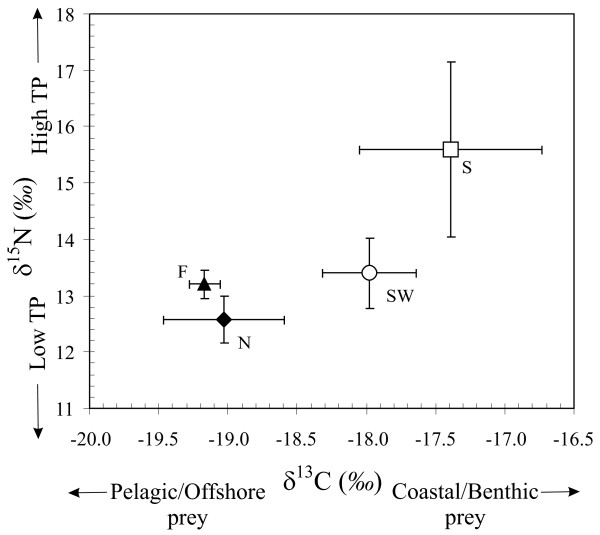
**Stable isotopes of carbon (δ^13^C) and nitrogen (δ^15^N) (mean ± SD) for harbour porpoises from the southern waters (S), the southwest coast of Norway (SW), Nordland (N), and Finnmark (F)**. TP: Trophic Position.

Stable-carbon and stable-nitrogen isotope measurements and trace element concentrations are shown by area respectively in Figure 2 and in Table 2.

**Table 2:** Trace element concentrations (μg·g^-1 ^dw) assayed in liver (for Zn, Cu, Fe, Se, Hg) and kidney (for Cd) of harbour porpoises from Scandinavian waters.

	Region	n	Mean ± SD	Range	ANOVA/ANCOVA(*)		
Zn	southern	13	118 ± 48	75 – 261	F_3, 28_: 2.6	p = 0.071	a
	southwest	9	103 ± 22	69 – 140			a
	Nordland	7	94 ± 8	86 – 109			a
	Finnmark	3	74 ± 2	74 – 76			a
Cu	southern	13	23.8 ± 4.7	15.4 – 33.2	F_3, 28_: 2.4	p = 0.089	a
	southwest	9	26.3 ± 7.1	17.2 – 39.1			a
	Nordland	7	25.5 ± 4.6	19.7 – 34.1			a
	Finnmark	3	17.7 ± 5.4	12.1 – 22.9			a
Fe	southern	13	1330 ± 480	860 – 2590	F_3, 28_: 0.5	p = 0.665	a
	southwest	9	1230 ± 310	810 – 1770			a
	Nordland	7	1100 ± 240	790 – 1440			a
	Finnmark	3	1220 ± 260	980–1500			a
Se*	southern	12	9.8 ± 7.3	4.3 – 31.6	F_3, 27_: 4.6	p = 0.010	a
	southwest	9	17.7 ± 8.0	6.1 – 33.5			b
	Nordland	7	14.8 ± 5.7	5.9 – 24.9			b
	Finnmark	3	6.7 ± 0.9	5.8 – 7.5			a
Hg*	southern	11	10.3 ± 10.9	4.1 – 42.8	F_3, 26_: 20.1	p < 0.001	a
	southwest	9	19.7 ± 9.8	6.1 – 31.6			a
	Nordland	7	15.5 ± 8.3	3.8 – 26.5			a
	Finnmark	3	0.8 ± 0.1	0.6 – 0.9			b
Cd*	southern	12	1.0 ± 0.8	0.1 – 2.3	F_3, 26_: 19.6	p < 0.001	a
	southwest	8	5.6 ± 2.5	2.7 – 10.4			b
	Nordland	7	9.2 ± 5.2	3.6 – 15.9			c
	Finnmark	3	5.7 ± 2.2	3.4 – 7.6			bc

Geographic differences among groups are indicated: areas followed by the same letter are not significantly different. n = number of samples.

### Principal components analysis (PCA)

**Figure 3:**
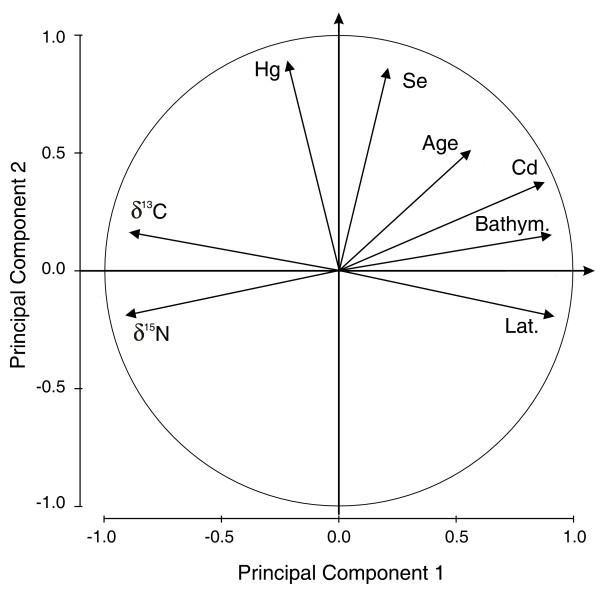
**Correlation biplot showing the distribution ("loadings") of the original 8 variables plotted on principal components 1 and 2**. The correlation between variables and between variables and principal components is given by the angle between the vectors. A right angle indicates no correlation whereas an acute or obtuse angle indicates a positive or negative correlation respectively. The length of the vector's projection on the component's axes reflects its contribution to the principal component. Because the analyses have been performed on the correlation matrix, vector lengths range between 0 and 1 as illustrated by the unit radius (the unitary correlation circle). Bathym.: Bathymetry; Lat.: Latitude.

An initial PCA produced a Kaiser-Meyer-Olkin's measure of sample adequacy (KMO) of 0.69, indicating that the use of a PCA was appropriate. However, Zn, Cu, and Fe had individual KMO and communalities < 0.50 and thus, were excluded from the analysis. Their removal resulted in a KMO of 0.77, low correlations in the residuals matrix, and significant loadings of all variables on at least one principal component (Figure 3).

The first 2 principal components (PCs) extracted had eigenvalues greater than 1.0 and accounted for 81% of the total variation present in the data set. Principal component 1, which explained 57% of the variance, indicated that increases in Cd concentrations were associated with the bathymetry, the latitude and with decreases in δ^13^C and δ^15^N values (Figure 3). Principal component 2, which accounted for 24% of the variance, showed a strong positive relationship between Hg and Se levels (Figure 3). Age was associated to a lesser extent with both factors and indicated a relationship with Cd concentrations, but not with Hg concentrations.

### Inter-site variation

The age of harbour porpoises (Table 1) did not differ significantly among regions (F_3, 27_: 2.8, p = 0.06). Carbon and nitrogen stable isotope measurements (Figure 2) differed significantly among sampling sites (Table 3). Regarding trace elements, concentrations in hepatic Se, Hg, and renal Cd differed significantly among sites, while Zn, Cu, and Fe concentrations showed no significant differences among sites (Table 2).

**Table 3:** Stable-carbon (δ^13^C) and stable-nitrogen (δ^15^N) isotope values (‰) in muscle tissue of harbour porpoises from Scandinavian waters.

	Region	n	Mean ± SD	Range		ANOVA	
δ^13^C	southern	13	-17.8 ± 0.7	-18.4 – -16.2	F_3, 28_:	p < 0.001	a
	southwest	9	-18.0 ± 0.3	-18.3 – -17.3			b
	Nordland	7	-19.0 ± 0.4	-19.5 – -18.2			c
	Finnmark	3	-19.2 ± 0.1	-19.3 – -19.1			c
δ^15^N	southern	13	15.6 ± 1.5	12.9 – 18.1	F_3, 28_:	p < 0.001	a
	southwest	9	13.4 ± 0.6	12.6 – 14.4			b
	Nordland	7	12.6 ± 0.4	12.1 – 13.1			c
	Finnmark	3	13.2 ± 0.3	12.9 – 13.4			bc

Geographic differences among groups are indicated: areas followed by the same letter are not significantly different. n = number of samples.

**Figure 4:**
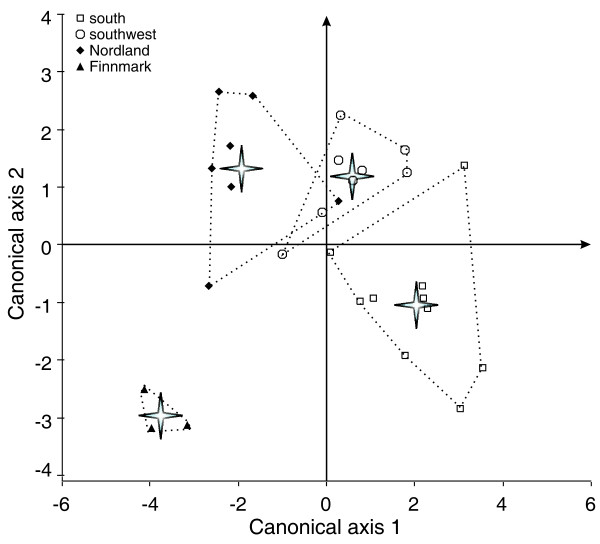
**Discriminant function scores for harbour porpoises from the four sampling areas (southern, southwest, Nordland, and Finnmark) plotted on the first two canonical axis**. Group centroids are indicated with a grey star.

The discriminant function (DF) analysis yielded three discriminant functions, with the first two accounting for 94% of the variation between groups (Table 4). There were significant differences among group centroids for the first two functions (DF1: Wilks' Lambda; χ92
 MathType@MTEF@5@5@+=feaafiart1ev1aaatCvAUfKttLearuWrP9MDH5MBPbIqV92AaeXatLxBI9gBaebbnrfifHhDYfgasaacH8akY=wiFfYdH8Gipec8Eeeu0xXdbba9frFj0=OqFfea0dXdd9vqai=hGuQ8kuc9pgc9s8qqaq=dirpe0xb9q8qiLsFr0=vr0=vr0dc8meaabaqaciaacaGaaeqabaqabeGadaaakeaaiiGacqWFhpWydaqhaaWcbaGaeGyoaKdabaGaeGOmaidaaaaa@3089@ = 78.6; p < 0.001; DF2: Wilks' Lambda; χ42
 MathType@MTEF@5@5@+=feaafiart1ev1aaatCvAUfKttLearuWrP9MDH5MBPbIqV92AaeXatLxBI9gBaebbnrfifHhDYfgasaacH8akY=wiFfYdH8Gipec8Eeeu0xXdbba9frFj0=OqFfea0dXdd9vqai=hGuQ8kuc9pgc9s8qqaq=dirpe0xb9q8qiLsFr0=vr0=vr0dc8meaabaqaciaacaGaaeqabaqabeGadaaakeaaiiGacqWFhpWydaqhaaWcbaGaeGinaqdabaGaeGOmaidaaaaa@307F@ = 38.2; p < 0.001) (Figure 4). Cross-validation using a jack-knife analysis showed a total of 89% of harbour porpoises were correctly assigned, with each group having at least 80% correct classification (Table 5). The stepwise procedure selected δ^13^C, Cd and Hg concentrations as significant predictors of group discrimination. Concentrations in Zn, Cu and Fe were removed from the model because of low discriminatory power, while δ^15^N values and Se concentrations were left out of the model due to colinearity respectively with δ^13^C values and Cd concentrations, and with Hg concentrations (see the PCA, Figure 3).

**Table 4:** Discriminant function (DF) loadings for each variable sorted by their importance in discriminating harbour porpoises by sampling group.

Variable	DF1	DF2
**δ^13^C**	**0.83**	-0.12
**Cd**	**-0.59**	**0.51**
**δ^15^N**	**0.58**	-0.01
**Hg**	0.37	**0.92**
**Se**	0.11	**0.54**
Zn	-0.04	-0.28
Cu	-0.34	0.10
Fe	-0.43	0.10
Eigenvalue	4.6	2.5
% of var.	61	33

Variables and coefficients in **bold **are those that had the strongest effect on discrimination (r > 0.5). Eigenvalues and the percentage of total variance (% of var.) among groups accounted for by the canonical variables are also given.

**Table 5:** Cross-validated counts for harbour porpoises from the discriminant analysis based on their isotopic and elemental compositions.

**Observed group**	**Assigned to group**			
	southern	southwest	Nordland	Finnmark
southern	80% (8)	20% (2)	0% (0)	0% (0)
southwest	0% (0)	100% (8)	0% (0)	0% (0)
Nordland	0% (0)	14% (1)	86% (6)	0% (0)
Finnmark	0% (0)	0% (0)	0% (0)	100% (3)

Columns show original sampling localities; rows show the percent and the absolute number (in parentheses) of samples classified into each region based upon canonical scores.

The structure coefficients (Table 4) indicated that the descriptors that best accounted for the among-group variation along the DF1 were δ^13^C values, followed by renal Cd levels and δ^15^N values, and along the DF2, the hepatic Hg concentrations, followed by Se and Cd levels. Harbour porpoise DF1 scores differed significantly among all sites except between Nordland and Finnmark porpoises (ANOVA on DF1 scores, F_3, 24_: 36.7, p < 0.001, Table 6). The individual scores and the group's centroids along DF1 increased gradually from northern to southern areas (Figure 4), indicating an increase in δ^13^C and δ^15^N values associated with a decrease in Cd concentrations from northern to southern porpoises. The second discriminant function significantly separated Finnmark harbour porpoises from all other groups. Southern, southwest and Nordland were not significantly different from each other (ANOVA on DF2 scores, F_3, 24_: 20.1, p < 0.001, Table 6). Finnmark harbour porpoises scored significantly lower than the others because of very low Hg and Se concentrations (Table 2). Porpoises from southern waters displayed intermediate DF2 scores with slightly lower Hg and Se concentrations than those from the southwest coast of Norway and from Nordland (Table 2).

**Table 6:** Post-hoc comparison (Tukey's tests) on harbour porpoise scores of the first and second discriminant function (respectively below and above the diagonal).

	southern	southwest	Nordland	Finnmark
southern	-	***	***	*
southwest	*	-	ns	***
Nordland	***	***	-	***
Finnmark	***	***	ns	-

* p < 0.05; ** p < 0.01; *** p < 0.001; ns: not significant.

## Discussion

The eight elements analysed in Scandinavian porpoise' tissues displayed three kinds of spatial variation: 1) those that displayed no significant variation among groups (Zn, Cu, and Fe concentrations); 2) those that varied among groups but without relationship to habitat characteristics (Hg and Se concentrations); and 3) those that varied geographically following a northward bathymetric gradient (δ^13^C, δ^15^N values and Cd concentrations).

Concentrations of all the essential elements, with the exception of Se, did not vary significantly among the four sampling sites and did not show any relationship with stable isotope compositions nor with the latitude and the bathymetry. Although the lack of significant differences among sites can partly be related to the small sample size, it is also likely that homeostatic regulation of these element levels in organism attenuate markedly the geographic variations.

We hypothesised that non-essential elements would be more useful as ecological tracers than essential elements. However, Hg concentrations did not show any relationship either with the stable isotope compositions, or with latitude and bathymetry. Instead, Hg concentrations correlated highly with Se levels (Figure 3). This relationship is frequently attributed to a detoxification process of Hg widely reported in marine mammals [30-32]. It involves seleno-proteins that contribute to the demethylation of methyl-mercury and leads to the production of an insoluble mineral granule of mercuric-selenide (tiemmanite), which accumulates during the life span of the organism [26,33]. This detoxification process also explains why Se levels did not follow the spatial pattern we observed for other essential elements. Concentrations of hepatic Hg and Se reported in this study and the site differences we observed were in general similar to those reported previously for porpoises from the Norwegian waters [34]. An exception was the three porpoises from Finnmark, which had lower hepatic Hg and Se concentration than previously reported. This may be an artefact of their younger age relative to porpoises analysed by Teigen et al. [34] (2.3 ± 0.3 yrs *vs *4.1 ± 2.8 yrs). The lower Hg and Se levels found in porpoises from the southern waters compared to those from the southwest coast and Nordland (Table 2) may also be due to slight differences in age structure of porpoises making up these samples (Table 1), although differences in age were not significant among regions. Thus, the spatial variation in Hg and Se concentrations may be more related to intrinsic properties and/or physiological processes rather than to ecological or environmental variation. Furthermore, Hg is derived mainly from fish regardless of the species [35] and given that these items are the main components of porpoise diet in all areas [29], this element may have no discrimination power among food sources. All these factors limit the use of Hg and Se to track ecological variation. In contrast, Cd concentrations correlated highly with stable isotope ratios, with the bathymetry and with the latitude (Figure 3), which indicates a strong relationship with the feeding ecology and with the environmental characteristics.

The spatial variation in the carbon and nitrogen isotope composition and in Cd levels accounted for most of the variation present in the data set. Nitrogen stable isotopes are generally used as a continuous indirect estimate of trophic position of consumers in food webs. However, it is impossible to define trophic position without an appropriate isotopic baseline because the pools of nitrogen that support marine food webs have different δ^15^N values [36], and because primary producers use different sources of nitrogen in different areas and seasons [37,38]. Therefore, the δ^15^N baseline is highly variable spatially and temporally, and measuring the production-weighted mean δ^15^N of phytoplankton on a large scale is logistically and analytically challenging. Jennings and Warr [39] studied the δ^15^N spatial variation in a suspension feeding bivalve molluscs in the north-east Atlantic to assess time-integrated spatial variation in the δ^15^N of primary producers and its impact on δ^15^N values of consumers. This study showed significant ^15^N enrichment with decreasing salinity and depth, and increasing temperature. This basal variation is quite similar to that reported for stable carbon isotopes and thus leads to the enrichment of the heavier stable isotopes toward coastal shallow waters [15-17]. This base δ^15^N variation accounted for most of the spatial variation observed in consumers, with the largest impact observed when comparing animals from shallow low salinity costal areas with deep high salinity sites [39]. Similarly, the present results showed that the greatest differences in porpoise δ^15^N values (~3‰) were between those from the southern waters, which are shallow and less saline, and those from deeper more saline waters of Nordland and Finnmark. This observation together with the correlated carbon and nitrogen stable isotope ratios, and bathymetry lead us to the conclusion that the variation in δ^15^N observed in porpoises merely reflects variation in basal δ^15^N rather than a trophic level variation.

Variation in carbon isotope composition among marine animals generally reflects differences in the isotope composition of marine plants between nearshore and offshore ecosystems [16,17,19]. Factors contributing to the base δ^13^C variation include differences in: (1) the carbon input from benthic macrophytes, such as the highly abundant kelp forest along the Norwegian coast that are significantly enriched in ^13^C relative to phytoplankton [16-19], (2) the species composition and growth rate of phytoplankton [40,41], and (3) the isotopic composition and concentration of dissolved CO_2 _[42-44]. In coastal and/or shallow waters, these factors lead to the production of relatively ^13^C enriched organic carbon at the base of the food web. In offshore areas, organic carbon at the base of the food web is more depleted in ^13^C due to limited nutrients, lower phytoplankton growth rate, and an absence of macrophytes. The significant ^13^C and ^15^N depletion observed in porpoises from southern toward northern waters (Figure 2) indicates a shift in harbour porpoise feeding habits from the North Sea to the Barents Sea that is strongly related to the local bathymetry (Figure 3): ^13^C and ^15^N depleted harbour porpoises from deep Nordic waters (Nordland and Finnmark) rely more on pelagic items of lower trophic level than those from the shallower southern waters within the North Sea (southwest and Skagerrak), which forage more on coastal or demersal items with a slightly higher trophic position. This change in porpoise feeding habits is fully consistent with the stomach content analyses [29,45]. Those studies showed a greater contribution of pelagic plankton-feeder fishes northwards (herring *Clupea harengus *and capelin *Mallotus vilosus*) *vs*. a more diverse diet in the southern waters composed of a wide range of fish species from benthic and pelagic habitats (*e.g*., saithe *Pollachius virens*, blue whiting *Micromesistus poutassou*, and pearlsides *Maurolicus mulleri*). This variation in diet probably reflects variation in food availability. It is currently assumed that harbour porpoises forage mainly within the first 200 m of the water column [46]. In Scandinavian coastal waters, depth varies greatly from shallow southern areas (generally less than 60 m in Danish and Swedish waters) to deeper northern areas where depth rapidly exceeds the preferred dive depth of harbour porpoise (Figure 1 and Table 1). Therefore, the higher bathymetry in Nordic waters reduces the availability of demersal prey species; the sea floor in the southern area will, on the other hand, be within reach for the most part [29].

The latitudinal cline of δ^13^C in porpoises (Figure 3) also suggests that a natural cline in stable isotope ratios may exist in Atlantic marine food webs, similar to that observed in the Pacific food webs [47,48]. Lower sea temperatures which increase the solubility and concentration of ^13^C-depleted CO_2 _in marine waters and decrease phytoplankton growth rates, may contribute to lower δ^13^C values at high latitude [19,43]. The Cd concentrations in porpoise kidney also displayed a north-south cline (Figure 3). The northward increase in Cd concentrations along the Scandinavian coast lend support to the hypothesis of a general "Cd-anomaly" in polar waters suggested by high levels of Cd reported in some polar invertebrates, such as crustaceans and molluscs, compared to their congeners in temperate waters [49-52]. The Cd levels increased significantly in δ^13^C and δ^15^N depleted porpoises coming from deep northern waters, revealing that animals in deep water feed on ^13^C- and ^15^N-depleted pelagic preys that are Cd-enriched compared to their congeners in shallow coastal waters of the south of Norway. Bustamante et al. [51] observed that oceanic cephalopods had very high Cd levels in sub polar areas and suggested that they were one of the main vectors for Cd transfer to top marine predators in the north-east Atlantic. Offshore cephalopods are also ^13^C- and ^15^N-depleted compared to coastal ones [53]. The higher Cd levels and the isotopic signature of offshore foraging observed in harbour porpoise of deep northern waters (Nordland and Finnmark) are likely to result from feeding habit in pelagic waters with occasional consumption of cephalopods or other Cd-enriched invertebrates. However, the presence of cephalopods in harbour porpoise diet was not recorded by stomach content analyses along the Norwegian coast [29,45], but has been reported in other areas [9,54]. This may originate from differences in integration time between the types of data used. Stomach contents provide dietary information for some days prior to the capture of animals and are unable to integrate seasonal variation. On the other hand, stable isotope ratios and trace element concentrations can retain dietary information on larger time scale that depends upon the elemental turnover rates of the tissue. In mammalian muscle, carbon and nitrogen stable isotopes retain information of previous feeding for some months [55-57], while the residence time of Cd in mammalian kidney is on the order of years [58]. The elemental profile in a given porpoise is thus an integration of the food assimilated over the season for stable isotopes (medium term) and over years for Cd (long term). The harbour porpoises analysed here and those studied for their stomach contents [29,45] were by-caught in spring. This time corresponds to the spawning period for several fish species, such as herring [59] and capelin in northern Norway and Russia [60], which congregate in large number off the Norwegian coast and then disperse offshore. Therefore, these species are widely available for predators, as evidenced by stomach contents of harbour porpoise [29,45]. The oceanographic constraints, together with the seasonal variability of the preferred prey availability could force porpoises, and especially those from northern waters, to forage in pelagic waters and include opportunistic prey such as cephalopods in their diet.

Concerns about porpoise populations' sustainability has arisen from high mortality rates reported in commercial gillnet fisheries [3], and was the impetus for studies of population structure (reviewed by [61]). Based on assumed porpoise distribution and abundance plus oceanographic features, three populations of harbour porpoises have been recognised along the Scandinavian coast (Figure 1) [62]: (1) the Kattegat Sea and adjacent waters, (2) the North Sea, and (3) north Norway/Barents Sea, with the putative division between the two latter located on the west coast of Norway at 66°N [63] (Figure 1). However, this proposed population structure was not supported by genetic analyses [64,65]. These authors suggested that the lack of genetic differentiations along the Norwegian coast may be a result of the recent recolonisation of the extreme north-east Atlantic since the last ice age, *ca*. 10 000 years ago, and/or from high gene flow within the region. In contrast, a finer population structure was suggested from the observation of significant differences in levels of radioactive caesium [28] and organic pollutants [66] between sampled groups along the Norwegian coast. The present results are consistent with these studies, and show that porpoises from the southern waters, from the southwest coast of Norway, and from more northern waters (Nordland and Finnmark) have significant differences in stable isotope compositions and Cd concentrations. Based on their elemental signature, almost all porpoises were correctly assigned to their *a priori *group, except three porpoises (two from the southern waters and one from Nordland), which were assigned to the neighbouring southwest site (Table 5). However, the significant difference in elemental signatures among porpoise sampling sites does not explicitly imply that ecologically or demographically distinct groups exist in Scandinavian waters. Although it often considered useful to define groups for management purposes, it should be noted that any arbitrary grouping of samples with sufficiently different averages of variables autocorrelated spatially could be found to be statistically different. Given that these statistical differences may reflect only sample clustering instead of biological reality, it is perhaps better, therefore, not to over-emphasise a finding that *a priori *groups are different. As stable isotopes and Cd provide information in the medium to the long term respectively, the spatial variation in the biochemical tracers observed here shows that harbour porpoises experience different ecological regimes within the year along the Scandinavian coast, adapting their feeding habits to local oceanographic conditions without performing extensive migration. This fact is supported by satellite tracking of porpoises tagged in Inner Danish waters and in Skagerrak/North Sea for up to 355 days [67] and in the Barents Sea throughout the breeding season [68], where animals restricted their movement preferentially to their respective areas. There was no overlap in the home range of adult porpoises tagged in the Inner Danish waters and in Skagerrak/North [67], likewise the seven satellite-tagged porpoises did not moved west of 29°E in the Barents Sea [68].

## Conclusion

Among the trace elements analysed in this study, only Cd, with its oceanic specific origin in the food web, was found to be useful as an ecological tracer. The other trace elements were not, probably because of physiological regulation and/or few specific source in the food web. Due to the longer integration time of Cd in animal tissue, this element complements the medium-term information provided by the stable isotope measurements. The signature of naturally occurring stable carbon and nitrogen isotopes and Cd levels in Scandinavian harbour porpoises were strongly related to the local bathymetry and latitude. In agreement with published stomach content analyses, the variation in elemental signatures suggested that harbour porpoises experience different ecological regimes during the year along the Scandinavian coast, adapting their feeding habits to local oceanographic conditions. This also suggests that harbour porpoises do not perform extensive migration along the Scandinavian coast, despite their potential to be highly vagile.

## Methods

### Sampling

In order to reduce potentially confounding effects linked to the individual physiological properties on elemental measurements, we have analysed harbour porpoises of relatively similar ages among regions (Table 1) and in good body condition according to the assessment made during the necropsy. Twenty adult porpoises were obtained from incidental catches in gillnet fisheries along the Norwegian coast between March and April 2000 and twelve individuals from Danish waters analysed in Das et al. [69] were also integrated into this study. Overall, thirty-two by-caught harbour porpoises (23 females, 9 males) were analysed from 4 different regions along the Scandinavian coast (Figure 1): southern waters (Skagerrak and adjacent areas) (n = 13), southwest coast of Norway (North Sea; n = 9), Nordland (Norwegian Sea; n = 7), and Finnmark (Barents Sea; n = 3). Age was determined by counting the dentinal growth layers of the teeth [70]. Samples of liver, kidney and muscle were stored at -20°C until analysed.

### Stable isotope ratios measurements

Analyses were performed on muscle tissue from which lipids were extracted by repeated rinsing with chloroform-methanol (2:1, v:v). After drying at 50°C for 48 h, samples were ground into a homogenous powder. Isotopic analyses were performed using a V.G. Optima (Micromass) mass spectrometer coupled to a Carlo Erba elemental analyser (C-N-S NA 1500NC, Fisons) with a precision of 0.3‰ for both ^13^C and ^15^N. Stable isotope abundance is expressed in delta notation (δ in ‰), relative to a standard (the VPDB, *Vienna PeeDee Belemnite*, and atmospheric nitrogen respectively for ^13^C and ^15^N) according to the following equation:

δ*X=(RsampleRstandard−1)×1000
 MathType@MTEF@5@5@+=feaafiart1ev1aaatCvAUfKttLearuWrP9MDH5MBPbIqV92AaeXatLxBI9gBaebbnrfifHhDYfgasaacH8akY=wiFfYdH8Gipec8Eeeu0xXdbba9frFj0=OqFfea0dXdd9vqai=hGuQ8kuc9pgc9s8qqaq=dirpe0xb9q8qiLsFr0=vr0=vr0dc8meaabaqaciaacaGaaeqabaqabeGadaaakeaaiiGacqWF0oazcqGGQaGkcqWGybawcqGH9aqpcqGGOaakdaWcaaqaaiabdkfasnaaBaaaleaacqWGZbWCcqWGHbqycqWGTbqBcqWGWbaCcqWGSbaBcqWGLbqzaeqaaaGcbaGaemOuai1aaSbaaSqaaiabdohaZHqaciab+rha0jab+fgaHjab+5gaUjabdsgaKjabdggaHjabdkhaYjabdsgaKbqabaaaaOGaeyOeI0IaeGymaeJaeiykaKIaey41aqRaeGymaeJaeGimaaJaeGimaaJaeGimaadaaa@50C9@

where X corresponds to ^13^C or ^15^N and R is the ratio ^13^C/^12^C or ^15^N/^14^N. Reference materials used were IAEA-N1 (δ^15^N = 0.4 ± 0.2‰) and IAEA CH_6 _(sucrose) (δ^13^C = -10.4 ± 0.2‰).

### Trace element analyses

Concentrations of Zn, Cu, Fe, total Hg and Se were determined in samples of liver, and Cd concentrations in kidney.

#### Zn, Cu, Fe and Cd analyses

Samples were weighed, dried for 48 h at 90°C, and digested with a solution of nitric acid (Merck 456) (1:3, v:v) slowly heated to 100°C until complete digestion. Concentrations, expressed as micrograms per gram of dry weight (μg·g^-1 ^dw), were assayed with an inductively coupled plasma-atomic emission spectrometer (ICPS: ARL-3510).

Parallel to the sample analysis, a set of certified sample material (CRM 278 Community Bureau of Reference, Commission of the European Communities) was also analysed to ensure reliability. Recoveries ranged from 88 ± 1% to 99 ± 2% for Zn, Cu and Fe and Cd. Metal specific detection limits were respectively for Zn, Cu, Fe and Cd: 0.2, 0.6, 0.2, and 0.4 μg·g^-1 ^dw.

#### Total Hg and Se analyses

Total Hg concentrations (in μg·g^-1 ^dw) were determined by cold vapour atomic absorption spectrometry on a Perkin-Elmer Coleman Mas-50 Mercury Analyser (wavelength 253.7 nm). Approximately 0.5 grams of wet weight (ww) tissue were mineralised with 4 ml HNO_3 _(Merck 456) and 1 ml H_2_O_2 _(Merck 7210) heated for 45 min. in a microwave oven (MLS-1200 MEGA). Prior to the analysis, the digested solution containing oxidized mercury was acidified with 2 ml H_2_SO_4 _97% (Merck 731) and reduced into volatile elemental mercury with 2 ml SnCl_2 _2% (Merck 7810) [71].

Part of the mineralised solution obtained from mercury analyses was used in the selenium analysis. The selenium in solution was complexed to EDTA and 2-diaminonaphtalene. The complex was then extracted by cyclohexane according to a protocol adapted from [72]. Selenium analyses were performed by fluorimetry on Perkin-Elmer Luminescence Spectrometer LS50B. Excitation and emission wavelengths were set at 364 and 523 nm respectively with an emission slit of 2 mm.

Quality control measurements for total Hg and Se included replicate analysis resulting in coefficients of variation < 10% and analysis of certified material (DORM-1, NRC Canada and CRM 278). Recoveries were 82 ± 6% and 108 ± 4% for Hg and Se. Hg and Se detection limits were respectively 0.02 and 0.7 μg·g^-1^dw.

### Habitat characteristics

Local bathymetric data were extracted from the ETOPO2 dataset available on the U.S. National Geophysical Data Centre (NGDC) [73]. In order to define local habitat characteristics, we calculated the mean depth (± SD) within a radius arbitrarily set at 100 km around each sampling locality using the Spatial Analyst extension in ArcGIS™ 8.2 (ESRI^®^) (Table 1).

### Statistical analyses

We analysed the relationship between the stable isotope values (δ^13^C and δ^15^N) and the trace element concentrations (hepatic Zn, Cu, Fe, Hg, Se, renal Cd) with regard to local bathymethry, latitude of sampled localities, and age of harbour porpoises using a principal components analysis (PCA). The analysis was conducted on the correlation matrix to remove the influence of descriptors' dimensional heterogeneity [74]. Only principal components with eigenvalues greater than 1.0 were retained. After a first run, the explanatory power of the PCA and the relevance of each variable introduced into the analyses were assessed by the Kaiser-Meyer-Olkin's measure of sample adequacy (KMO) calculated for all variables and on each variables separately. KMO values vary between 0 and 1. Only variables with KMO values greater than 0.5 were retained for a second PCA as they indicated interdependence of variables and appropriateness of their inclusion in the analysis [75]. Finally, a varimax rotation was applied to simplify the final interpretation of principal components.

The differences in element levels among sampling localities were first tested using a univariate analysis of variance (ANOVA) followed by a post-hoc Tukey's test when appropriate. Because Cd and Hg are known to accumulate with age [26,33], an analysis of covariance (ANCOVA), with age as covariate, was used instead of the ANOVA. Geographic variation in element levels was further investigated using a discriminant function analysis (DFA) in order to test whether porpoises from the four localities were significantly different, and to identify the variables that contributed most strongly to their separation. The DFA was performed on the two stable isotopes (δ^13^C and δ^15^N) and the six trace elements (hepatic Zn, Cu, Fe, Se, Hg, and renal Cd) using a forward stepwise procedure and a criterion of p = 0.05 and p = 0.10 respectively to add and remove variables from the analysis. Cross-validation using a jack-knifed analysis was applied to verify the accuracy of the DFA. Since standardized canonical coefficients may cause problems of interpretation when descriptors are correlated, we have used the structure coefficient (*i.e*. the correlation ('loadings') between predictors and discriminant function) to assess the contribution of descriptors to the variability among groups [76].

All distributions were log_*e *_transformed to better approximate the homogeneity of variance and assumptions of normality for the tests performed [74]. To avoid problems in transforming negative δ^13^C values, we added the maximal absolute values recorded plus one to all δ^13^C values. Statistical analyses were conducted using SPSS statistical package, version 10.0.

## Authors' contributions

MCF, KD and JMB conceived and designed the study. MCF carried out the laboratory analyses, collected and analysed the data. MCF interpreted the results with help from KD, KAT, GL, SG. MCF wrote the manuscript with help from KD, KAT, and GL. US and KAT made the samples available for this study. All authors read and approved the final manuscript.
